# Early Findings on the Effectiveness of Novel Awakening Stimuli for Patients with Congenital Central Hypoventilation Syndrome

**DOI:** 10.3390/s25061759

**Published:** 2025-03-12

**Authors:** Silvia Rapella, Caterina Piazza, Francesco Morandi, Alessandro Carcano, Cinzia Arzilli, Niccolò Nassi, Igor Catalano, Francesca Formica, Emilia Biffi

**Affiliations:** 1 Scientific Institute, IRCCS Eugenio Medea, 23842 Bosisio Parini, LC, Italy; silvia.rapella@lanostrafamiglia.it (S.R.); caterina.piazza@lanostrafamiglia.it (C.P.); francesca.formica@lanostrafamiglia.it (F.F.); 2 Italian Association for Congenital Central Hypoventilation Syndrome (AISICC), 50126 Florence, FI, Italy; morandifrancesco55@gmail.com (F.M.); carcaalex@gmail.com (A.C.); 3 Meyer Children’s Hospital IRCCS, 50139 Florence, FI, Italy; arzillicinzia@hotmail.it (C.A.); niccolo.nassi@meyer.it (N.N.); 4 Pediatric Palliative Care, Pain Service, Department of Women’s and Children’s Health, University of Padua, 35128 Padua, PD, Italy; igor.catalano@vidas.it

**Keywords:** congenital central hypoventilation syndrome, assistive device, desaturation episodes, arousal

## Abstract

Congenital Central Hypoventilation Syndrome (CCHS) is a rare disorder that impairs autonomic breathing control, leading to alveolar hypoventilation and sometimes to central apnoea, predominantly during sleep. Patients typically require nocturnal ventilatory support and alarms to prevent life-threatening desaturation events. However, current alarm systems integrated into pulse oximeters do not provide adequate assistance at home. To address these limitations, we developed an assistive device with customizable multisensory stimulation that activates based on the severity and duration of desaturation episodes. In a multicenter clinical trial involving 4 children and 11 young adults with CCHS, we assessed the device’s effectiveness and the role of arousals over three nights: one baseline and two test nights. The results showed that the device significantly improved awakening rates and enabled faster recovery from desaturations in young adults. However, no such improvements were observed in children compared to the baseline. Arousal events and sleep efficiency were unaffected by the device in both groups. These findings suggest that the device can enhance the safety and autonomy of young adults with CCHS but may be more effective in alerting caregivers in pediatric cases than directly waking children. Further studies are needed to refine its application across different age groups, given the limited sample size.

## 1. Introduction

Congenital Central Hypoventilation Syndrome (CCHS), also referred to as Ondine’s curse, is a rare genetic disease characterized by impaired breathing control associated with Autonomic Nervous System (ANS) dysfunction whose incidence is estimated at around 1/200,000 live births [1]. A definitive diagnosis of CCHS is made by confirming PHOX2B gene mutation; the majority of patients with CCHS have polyalanine repeat mutations (PARMs), while approximately 10% of patients are heterozygous for non-polyalanine repeat mutations (NPARMs) [2]. CCHS is mainly characterized by abnormally reduced or absent ventilatory responses to hypercapnia and hypoxia during sleep.

Since there is no definitive therapy for this disease, patients with CCHS are assisted, predominantly during sleep, using diaphragm pacing, non-invasive ventilation (NIV) or invasive ventilation with tracheostomy [1,3,4]. Furthermore, their blood oxygenation is monitored throughout the whole night by means of non-invasive pulse oximeters that measure peripheral blood oxygen saturation (SpO_2_) according to the photoplethysmography principle, with a low-intensity infrared (IR) sensor usually located on a finger to assess potential decreases in oxygen levels in body tissues [5]. If low oxygen levels are detected by the pulse oximeter, the acoustic alarm integrated into the device signals the dangerous situation and wakes up the patient or alerts the caregiver [6]. The patient’s awakening allows the recovery of the correct saturation level after a period of time that depends on the initial desaturation level. In contrast, without a wake-up stimulus or intervention, the CO_2_ anesthesia can cause neurological damage and also lead to patient death. Currently, the only alarm systems available for patients with CCHS are those integrated into pulse oximeters; these alarms trigger every time the SpO_2_ level falls below a safety threshold. Since patients may have reduced or an absent perception of audio alarms while asleep, a caregiver or a parent is needed all night long to serve as a supervisor and to take proper action if the situation worsens [7]. Sometimes oxygen saturation may fall below the threshold and return to normal levels within a few seconds while the patient is asleep, e.g., in case of sensor movement or an artefact in SpO_2_ measurements. This situation, although it is no real danger for the patient, causes the alarm to sound, as the pulse oximeter does not take the time variable into account. Therefore, during the night, the pulse-oximeter alarm may activate several times for borderline situations and false alarms. This causes a tendency to lower the warning threshold values to not disturb the patient’s or caregiver’s sleep [8]. Another problem could be related to habituation: both patients and caregivers can become less responsive to frequent and repeated alarm stimuli. Thus, it is tough for young adults with CCHS to achieve independence and live on their own because of the risk that they doze off without wearing the ventilation mask or that, during the night, they do not hear the alarms and do not wake up.

A few studies have proposed the development of a system to wake up patients created specifically for this syndrome. Mayer et al. developed a mobile alarm system worn behind the ears that monitors the blood oxygen saturation level. If this drops below a pre-defined threshold, a loud acoustic signal is emitted to wake up the patient. However, the device has been tested only on one healthy subject; therefore, another study should be conducted to evaluate the usability of the prototype on people with CCHS [9]. Attali et al. tested on 20 healthy subjects and 20 patients with CCHS or Chronic Obstructive Pulmonary Disease (COPD) a device composed of a vibrating wristband and a phone application. In their study, they concluded that the use of vibrotactile stimulation alone is not able to awaken ventilator-dependent patients, including participants with CCHS. They suggest testing the efficacy of vibrotactile stimulation combined with an audio alarm [10]. In this context, our group developed an assistive device to improve assistance for patients with CCHS and help overcome the previously listed problem [6]. The device is coupled with a pulse-oximeter and carries out various multisensory stimulation strategies (acoustic, tactile, and proprioceptive stimuli) depending on the SpO_2_ blood concentration level and duration of desaturation. The idea is to lead to a certain awakening of the patient in critical conditions. In fact, the most intense sensory stimulation is used in life-threatening situations. If the device is able to awaken patients, it will increase the everyday safety and independence of teenagers and adults with CCHS. The device has been tested in two previous studies. In the first one, through the involvement of eight healthy subjects, it was demonstrated that the device works correctly, guarantees rapid awakenings, and does not affect the user’s sleep [11]. In the other one, the device was tested on a group of five individuals with CCHS, and it was found to be relatively effective in inducing wakefulness without impacting sleep quality. However, the results are considered preliminary due to the small sample size, which limited the ability to extensively test each actuator on all participants [12].

Another aspect that was reported in [12] was the efficacy of the device in inducing arousals, defined as a rapid shift in the electroencephalography (EEG) frequency lasting at least 3 s but no more than 15 s [13]. This phenomenon has been studied in adult patients with breathing disorders, showing the importance of arousal for recovery after desaturations. Indeed, the arousal is thought to be a protective reflex against hypoxemia. However, the effect of arousals in children and adolescents is still debated [14,15,16,17], and there is very limited knowledge about arousals events and their effects on subjects affected by CCHS.

This manuscript describes the first attempt to clinically validate the device previously developed by our group [11], thus paving the way to its applicability as an assistive device for CCHS patients.

Specifically, this manuscript describes the results of a multicenter clinical trial aimed at testing the device on 15 patients with CCHS of different ages. Particularly, we aimed to (1) analyze its efficacy in waking them up, in reducing the duration of desaturation, and assess if multisensory stimulation affects patients’ sleep efficiency in comparison with typical night setup; (2) investigate if the presence of arousals on the EEG traces enables a faster recovery of the saturation level; (3) verify whether a stimulation device is generally superior to others in waking patients.

## 2. Materials and Methods

### 2.1. Participants

The study population comprised 15 patients with CCHS: 4 children (2 boys and 2 girls; age = 8 ± 4 years) and 11 young adults (3 boys and 8 girls; age = 22 ± 7 years). The patients were a subgroup of the cohort constituting the A.I.S.I.C.C. (Italian Association for Central Congenital Hypoventilation Syndrome) association. All patients breathed spontaneously without assistance during the daytime while needing invasive or non-invasive ventilator support during the night. Everyone has a genetically confirmed diagnosis of CCHS; the PHOX2B mutations include 14 cases with polyalanine repeat mutations (PARMs) and one with nonPARM (NPARM) frameshift mutations. The demographical and clinical characteristics of our cohort are summarized in Table 1.

Recruitment and testing were completed in three different Italian centers with expertise in the management of patients with CCHS: IRCCS E. Medea, Meyer Hospital, and the Pediatric Pain and Palliative Care Service of the University of Padua. The study was approved by the Ethic Committee of IRCCS Medea (PROT. NO 199/13-CE; date 9 April 2013) and conducted according to the guidelines of the Declaration of Helsinki. All participants were informed about the methodology and duration of the study, and written informed consent to participate was obtained from them or from their parents prior to inclusion in the protocol.

### 2.2. Device Description

**Figure 1:**
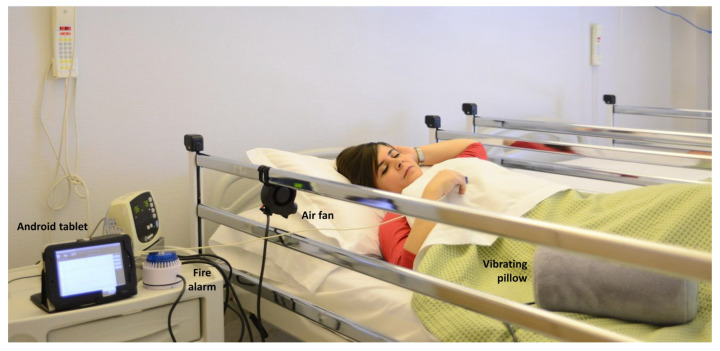
Assistive device with its actuators.

The assistive awakening device used in this trial is coupled with a pulse-oximeter and carries out various multisensory stimulation strategies, depending on the SpO_2_ level and the duration of the desaturation. The device has an Android tablet that dynamically monitors the SpO_2_ level as measured using a pulse-oximeter and activates, according to an ad hoc developed software, different actuators such as a piezoelectric buzzer (acoustic stimuli), a small air fan (tactile stimulation), a vibrating pillow (tactile/proprioceptive stimulation), and a fire alarm (strong acoustic stimulation) (Figure 1) [6]. All the actuators are commercial devices with the following characteristics:

- Air fan (Blower—Squirrel Cage, Sparkfun Electronics, Niwot, CO, USA): outlet diameter of 33 mm, rated airflow 16 cubic feet per minute (CFM) and a fan speed of 3000–3500 revolutions per minute (rpm).
- Vibrating massage pillow (Pillow-massager E.R. Rovera, Lissone, Italy): dimensions 30 × 16.5 × 13 cm.
- Piezoelectric buzzer (CET12A3.5, Sparkfun Electronics, Niwot, CO, USA): minimum Sound Pressure Level (SPL) of 85 dB.
- Fire alarm (ROSHNI LP (ROLP/SV, Fulleon, Cwmbran, UK)): acoustic stimulation of 100 dB.

To configure the device, four distinct severity levels (SLs) must be defined for each patient. These levels are determined by two key parameters: the maximum allowable time a patient can remain within a specific SpO_2_ range and the corresponding SpO_2_ value range. This dual approach enables the device to address both acute hypoxic episodes and prolonged mild hypoxia, which could pose a significant risk too. For each SL, one or more actuators can be configured, with adjustable stimulation intensity. This ensures complete customization of both the type and intensity of stimuli activated under various conditions. Additionally, the device records SpO_2_ levels, heart rate, timestamps, and alarm triggers at a sampling rate of 1 Hz. It also includes a user interface that facilitates real-time SpO_2_ monitoring and the efficient management of alarms [11,12].

The device was developed by using a co-design process involving patients, their families, and doctors from the Italian Association for Congenital Central Hypoventilation Syndrome (AISICC) to ensure compliance and ease of use.

### 2.3. Study Design

**Figure 2:**
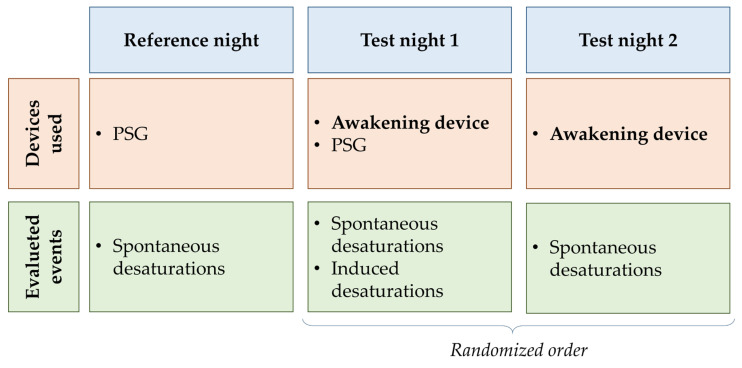
Study design and setting for each night. PSG: polysomnography.

The study protocol included 3 nights of testing per subject (Figure 2). During the first night (baseline night, B_N_), the standard monitoring system of each participant was used (i.e., proper assistive ventilation device and pulse-oximeter), and polysomnographic data were acquired. This night was used to perform a baseline evaluation of patient sleep and desaturation events. During the test nights (T_N1_ and T_N2_), the awakening device was connected to a pulse oximeter (Nonin Avant 9600, Nonin Medical, Plymouth, MN, USA) and desaturation events were recorded. The test nights were presented in a randomized way. T_N1_ differs from T_N2_ in that the subject also wears polysomnography sensors (Natus Embla N7000 or Natus Embletta X10 System, Natus Medical Incorporated, Middleton, WI, USA) to monitor electroencephalogram, respiratory flows, SpO_2_, heart rate, and to evaluate sleep efficiency. Since, during this night, the patient is in a controlled sleep laboratory setting, the clinician changes the ventilator parameter to induce at least one desaturation. Using this method, we could assess device activation in borderline situations.

In the two test nights, the SpO_2_ and time values associated with each SL were individually defined for each participant in agreement with his/her doctor. Only one actuator was activated for each SL. The fire alarm was always associated with SL4, which represents the most critical situation, while the other three actuators were randomly associated with each SL on each test night.

Each patient performed all acquisitions in the same center during three consecutive nights. One patient (S_008) completed only the reference night due to health problems not related to the clinical trial, while another (S_001) decided not to perform the reference data acquisition.

### 2.4. Data Acquisition

During the test nights, the different actuators were tested, thus providing us the parameters for identifying the efficacy of the device in waking up the participants, the effect of the stimulations on the participant’s sleep efficiency, and the effect of arousals on the recovery of the saturation level.

The awakening device was acquired, along with a sample rate of 1 Hz, SpO_2_ and beat-per-minute values, the time and type of actuators triggered, and the time the alarm remained active. The polysomnography sensors monitored the electroencephalogram, respiratory flow, SpO_2_, and heart rate. SpO_2_ values were also acquired through a standard pulse oximeter worn by each participant during all the nights.

### 2.5. Data Processing

Combining the information coming from the awakening device and the PSG, the following parameters were computed for each night and each actuator:

- number of spontaneous and induced desaturations that triggered the actuator;
- number of awakenings, time required for awakening, and time necessary for recovering the basal level of SpO_2_;
- number of arousals and time required for the saturation level to recover after the arousal (evaluated only during the nights with the PSG sensors);
- time required for SpO_2_ levels to return to baseline when no awakenings or arousals occurred;
- sleep efficiency.

Desaturation events were identified from the value of SpO_2_ detected by the pulse oximeter and recorded by the awakening device. These events were categorized as either spontaneous or induced, depending on their cause: events triggered by a clinician modifying ventilator parameters were classified as induced, while all others were considered spontaneous. For each participant, the total number of events, categorized as either spontaneous or induced, was calculated. It was decided not to subdivide the desaturations on the basis of severity level in order for the results to have statistical consistency. Additionally, SpO_2_ values were used to determine the recovery time following a desaturation for both types of events.

The offline analysis of the PSG recording, which was made by an expert clinician with several years of expertise, led to the identification of sleep stages, wake phases, and the presence of arousals during both spontaneous and induced desaturations. PSG data were analyzed with Somnologica Studio software (REM LOGIC 3 version 3.4.4.2413) by Embla N7000 and Embletta X10 System, and a scoring of the PSG tracks was performed using the age-appropriate criteria of the American Academy of Sleep Medicine [18]. Arousals were defined as a return to alpha or fast frequency EEG activity, well differentiated from the background, lasting at least 3 s but no more than 15 s [13]. The percentage of arousals was defined as the number of arousals over the number of non-awakenings events, calculated only for the nights for which these data (i.e., B_N_ and T_N1_) were available. An analysis of the PSG data was also carried out to calculate sleep efficiency (i.e., sleep/wake ratio based on the number of movements measured). Furthermore, combining the SpO_2_ data from the pulse oximeter and the EEG signal, the arousal effect was evaluated through a comparison between the time to recover basal SpO_2_ after arousal and the time to recover basal SpO_2_ if no arousal occurs.

To determine whether the patient was awake or asleep during the three nights, various types of data were analyzed. For B_N_ and T_N1_, EEG data collected from the polysomnography sensor were utilized. During the remaining night, the participant’s movements were visually assessed, and the moments when the patient manually silenced the alarm were taken as clear indications of wakefulness.

Then, we computed the device efficacy, defined as its ability to awaken the patient, as the number of awakenings over the total number of desaturation events (both induced and spontaneous), expressed as a percentage. By dividing the events based on the triggered actuator, the effectiveness of each actuator was likewise calculated.

Ultimately, we calculated the risk time, defined as the total time for which the patient’s life is in danger due to spontaneous desaturations. This parameter accounts for two scenarios: the time before awakening if the patient wakes up, or the time required to return to normal SpO_2_ levels if no awakening occurs, distinguishing between cases with arousal and those without. This time was computed when the device was available as well as without it (reference night).

### 2.6. Statistical Analysis

The data analysis included clustering the population into two subgroups: young adults and children (defined in the Section 2.1).

Concerning the statistical analysis, the normality of data distribution was computed using the one-sample Kolmogorov–Smirnov test. Since all the data were found to be not normally distributed, non-parametric statistical tests were used. A *p*-value < 0.05 was considered significant in all the tests.

First, the median and interquartile ranges (IQRs) of the percentages of awakenings were analyzed, comparing the values recorded during the test nights (T_Ns_, i.e., both T_N1_ and T_N2_) with those of the baseline night (B_N_). The same analysis was performed for the percentage of arousals. For both parameters, differences between the test nights and the baseline night were evaluated using the Wilcoxon signed-rank test. In the second step, the data on the percentage of awakenings and arousals were clustered based on the triggered actuator, and the differences in efficacy among actuators were assessed using the non-parametric Friedman test.

Furthermore, we compared the risk time with and without the device and the recovery time in relation to the presence/absence of the arousal effect. For these temporal parameters, only spontaneous desaturations were considered, as induced desaturations represent non-physiological and highly critical situations that do not reflect typical conditions. Medians and interquartile ranges (IQRs) were calculated for each temporal parameter in T_Ns_ and B_N_; differences between T_Ns_ and B_N_ were analyzed using the Mann–Whitney U test.

In the end, a comparison of sleep efficiency between T_N1_ and B_N_ was conducted using a Wilcoxon signed-rank test.

## 3. Results

Table 2 shows the total number of desaturation events (both induced and spontaneous) that occurred in the three nights, broken down by the type of actuator that is activated during the test nights.

### 3.1. Device Effectiveness: Awakenings, Risk Time, and Sleep Efficiency

Table 3 shows the percentage of awakening, the sleep efficiency, and the risk time during the test nights (with the device) and the baseline night (with the standard setting) on young adults and children. Interestingly, the percentage of awakenings on the test nights in young adults was statistically higher than on the night with the standard setting, with 25% (IQR = 21%) and 6% (IQR = 9%) of awakenings, respectively (*p* = 0.018). On the other hand, the device seems to have a reduced effect on children (N = 4, age = 8 ± 4), with 11% (IQR = 7%) of awakenings with the device and 3% (IQR = 5%) in the standard setting.

Sleep efficiency both in children and young adults was comparable in the nights with or without the use of the device. In fact, in young adults, the sleep efficiency was 71.3% (IQR = 7.2%) in T_N1_ and 79.3% (IQR =10.8%) in B_N_. In children, the sleep efficiency was equal to 88.2% in both nights, with IQR = 9% in T_N1_ and IQR = 11% in B_N_. Thus, the device does not affect the patient’s sleep more than the standard setting used by the patient. In general, children have higher sleep efficiency with respect to adults.

**Figure 3:**
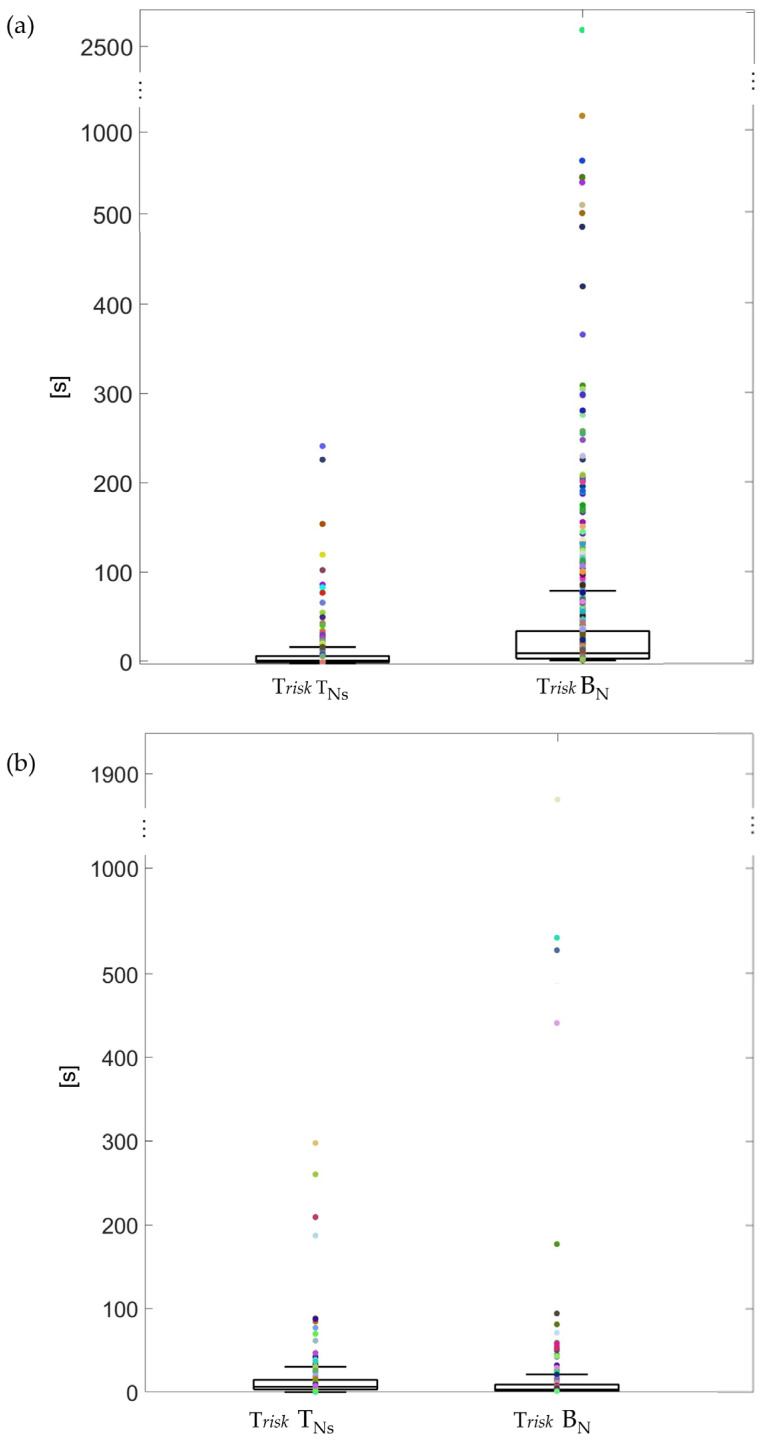
Risk time in young adults (**a**) and children (**b**). Each risk time is represented by a dot.

Considering the risk time, young adults exhibited a shorter risk time in T_Ns_ compared to B_N_ (*p* < 0.001), with most of the risk time values on test nights concentrating below 100 s, while spreading over 300 s on the baseline night (Figure 3a). This result was opposite for children (Figure 3b), who showed higher median values of risk time during the test night. Nevertheless, Figure 3b shows that most of the risk time values on test nights concentrated below 100 s, reaching 300 s in a few cases and 1900 s during B_N_. Furthermore, from a qualitative perspective, the children had a lower risk time in B_N_ with respect to young adults.

### 3.2. Arousal Effect

Table 3 shows that the percentage of arousals in young adults was higher in T_Ns_ with respect to B_N_: 23% (IQR = 23%) and 0% (IQR = 1%), respectively. These results were also confirmed in children with 29% (IQR = 15%) of arousals in T_Ns_ with respect to 0% (IQR = 1%) in B_N_. However, these differences were not statistically significant. Furthermore, young adults recovered significantly faster after an arousal (median = 4 s IQR = 6 s) caused by the activation of one of the stimulations performed by the device with respect to desaturations, during which the device did not cause arousals (median = 6 s, IQR = 25.5 s, *p* < 0.001). In contrast, in the children, an opposite trend was reported: the recovery time was 10 s (IQR = 19 s) and 3 s (IQR = 9 s), respectively (*p* = 0.004).

### 3.3. Differences Among Actuators

Table 4 shows the effects of the device on young adults and children divided by the type of actuators. The percentage of awakenings in young adults (N = 11, age = 22 ± 7) was 100% (IQR = 57%) for the fire alarm, while the vibrating pillow and the air fan showed lower and comparable efficacy. The worst results were obtained with the buzzer, with a percentage of awakenings of 23% (IQR = 67%). However, no significant difference was found among the stimulations. Unlike adults, in children, the most effective device was the vibrating pillow. The air fan had a slightly lower performance, while the buzzer and the fire alarm were not effective in waking them up.

Concerning the percentage of arousal in young adults, the fire alarm is the device that caused the highest number of arousal with respect to the number of non-awakenings equal to 66.7% (IQR = 50%). In contrast, the vibrating pillow was not effective in causing arousals even if no significant difference was observed.

Finally, in young adults, the fire alarm was the device associated with the shortest risk time, while, in children, the air fan and the vibrating pillow were the actuators that most effectively reduced it. However, no statistical difference emerged when comparing the risk time among actuators in both groups.

## 4. Discussion

Concerning the aim of the project, the device is efficient in waking up young adults with CCHS; however, it is not effective on children. In fact, in young adults, our device has a higher percentage of awakenings with respect to the standard setting.

In young adults, the device is efficient in cases of dangerous situations, since the fire alarm, which is the alarm set in situations with higher risk for the patient, usually wakes them. Also, the fire alarm seems to be the device with the shortest risk time. The other devices are less efficient as the percentage of awakening is below 50%. A previous study tested the efficacy of a vibrotactile device for CCHS or COPD patients (aged between 19 and 24 years old). They concluded that the vibrating stimuli itself is not efficient [10]. This is confirmed by our project since the vibrating pillow itself awakens young adults only in 39% of cases. However, in our study, the difference between the four actuators was not statistically significant; this could suggest that everyone is more responsive to a different type of stimuli, confirming the importance of the modularity of the proposed device, namely, that each patient can choose which device to associate with each SL based on his/her sensitivity. Moreover, the percentage of awakenings on the test nights was statistically much higher than on the night with the standard setting in young adults. These results confirm that our device improves the patients’ condition at night with respect to the common alarm integrated into the pulse oximeter. This is further confirmed by the significantly lower risk time when using the device with respect to the standard setting.

On the other hand, in children, the effectiveness in terms of both the number of awakenings and risk time is not confirmed. The percentage of awakening is not reduced with the device with respect to the standard setting, and it is possible to qualitatively appreciate the lower efficacy of all the actuators with respect to young adults. Also, the risk time is not lowered by the use of the device. However, risk time values were mainly concentrated under 100 s in both conditions. The fact that children do not react the same way as young adults to stimuli during sleep could be caused by a difference in sleep pattern that is characterized by important amounts of Slow Wave Sleep (SWS) that decrease sometime during puberty. Children have more periods of deep sleep, and it is, therefore, more difficult to wake them up [19,20], suggesting that the proposed device is not appropriate for this age group. Nevertheless, children do not live alone and do not need complete independence; therefore, the device could be a useful means to wake up their caregivers, reducing the habituation effect.

Regarding the effect of the device on arousals, the percentage of arousals corresponding to a desaturation and a stimulation activation is higher with respect to the same parameter when the standard setting is used both in young adults and children. However, the results of the statistical analysis show that the percentage of arousals in both groups does not depend on the used device. The importance of arousal for recovery after desaturation is thought to be a protective reflex against life-threatening hypoxemia in adults, while it is still debated in children and adolescents [14]. It is commonly stated in the literature that children with CCHS do not have arousal in response to endogenous hypoxemia and hypercapnia [17]. However, two studies contradict this conclusion. The first one tested the hypercapneic arousal responses of CCHS children (5.8 ± 1.2) during sleep while their home ventilators were used. During the night, a rapid increase in the inspired carbon dioxide tension was induced, and it has been shown that most CCHS children have arousal responses to hypercapnia [16]. Huang et al. studied the arousal associated with gas exchange abnormalities in CCHS patients (aged 13 ± 7 years). The study was performed by disconnecting the ventilator from the patients and evaluating the presence of arousal through overnight polysomnography. They concluded that children/youth with CCHS have frequent arousals associated with gas exchange abnormalities during sleep and that they occurred during active but not quiet sleep [15].

Another study conducted by McNamara et al. found that, in children (aged 1–14 years) with Obstructive Sleep Apnea (OSA), respiratory events were often terminated spontaneously, and that the duration of apnoea associated with arousal was longer compared with self-terminating apnoea [21]. This conclusion is supported by our findings in the children’s sample, where recovery time is extended when arousals occur compared to when they do not. Conversely, in young adults, recovery time is shorter with arousal than without. This may be due to an effect observed in some studies, where an improvement in ventilation during sleep has been seen in patients who actively move during rapid eye movement sleep or even when they are passively moved [22]. In our study, arousal is not the primary mechanism for recovery from desaturation, as only 23% of non-awakenings in children and 29% in young adults are associated with arousal. Nevertheless, this may have a protective effect in young adults.

Significantly, sleep efficiency was not affected by the presence of the device in both groups. In general, in healthy school-aged children, sleep efficiency is usually around 90% [23], while in healthy adolescents and adults, sleep efficiency is considered ‘good’ if it is higher than 85%. Therefore, in our population, this parameter is slightly below average during all the nights.

This study presents some limitations. The first one is related to the sample size, as the study was conducted only on 15 subjects, and the number of activations for certain actuators was relatively low. Particularly, the results obtained for the children’s group should be interpreted with caution, as they are based on tests conducted with only four subjects. Nevertheless, the target population for the device consists of adolescents and young adults. In the case of children, we anticipate that the device’s stimulations could be directed toward the caregiver, thereby supporting their assistive role. Additionally, the limited number of recorded desaturation events restricted the ability to discern differences across varying severity levels of desaturation. Moreover, a limitation of the device stems from the use of commercially available actuators for which not all the stimulation characteristics (e.g., frequency and amplitude of the vibrations) are known. Future studies should better characterize the effect of stimuli manipulation on the device’s effectiveness. Future developments of the device could leverage AI-based pattern recognition to improve its responsiveness to desaturation events, thereby enhancing its ability to dynamically adjust to individual responses. Moreover, the next step of validation will include the testing of the device at patients’ homes to see how it works in their familiar environment over an extended period. Furthermore, testing on the caregivers of children with CCHS will be performed to see if the device also elicits improvements in parents’ quality of life.

In conclusion, the awakening device is efficient on adolescents/youth affected by CCHS; however, it is less effective on children. Therefore, the device could be useful to wake up caregivers in response to child desaturation. The results suggest that the device proposed is effective in improving domiciliary care and the quality of life of young people who want more independence, even if it would be useful to collect more data since the patient sample size is quite small.

## Figures and Tables

**Table 1:** Participants’ characteristics and site of the testing procedure. NIV: non-invasive ventilation. Tracheo: tracheostomy. PPPCS: Pediatric Pain and Palliative Care Service of the University of Padua.

Subject	Gender	Age (Years)	Gene Mutation	Type of Ventilation	Testing Location
S_001	F	18	PHOX2B 20/27 PARM	NIV	IRCCS E. Medea
S_002	M	5	PHOX2B 20/27 PARM	Tracheo	IRCCS E. Medea
S_003	M	5	PHOX2B 20/26 PARM	NIV	IRCCS E. Medea
S_004	F	18	PHOX2B frameshift NPARM	NIV	IRCCS E. Medea
S_005	F	19	PHOX2B 20/26 PARM	NIV	IRCCS E. Medea
S_006	F	16	PHOX2B 20/27 PARM	Tracheo	IRCCS E. Medea
S_007	M	17	PHOX2B 20/26 PARM	NIV	IRCCS E. Medea
S_008	F	11	PHOX2B 20/29 PARM	Tracheo	PPPCS
S_009	M	28	PHOX2B 20/26 PARM	NIV	Meyer Hospital
S_010	F	26	PHOX2B 20/25 PARM	Pacing	Meyer Hospital
S_011	M	22	PHOX2B 20/26 PARM	NIV/pacing	Meyer Hospital
S_012	F	12	PHOX2B 20/32 PARM	Tracheo	PPPCS
S_013	F	25	PHOX2B 20/26 PARM	Pacing	IRCCS E. Medea
S_014	F	39	PHOX2B 20/25 PARM	NIV	Meyer Hospital
S_015	F	23	PHOX2B 20/25 PARM	NIV	Meyer Hospital

**Table 2:** Number of total desaturations during the baseline night and the test nights (divided by the actuators that were elicited).

	Total Number of Desaturations				
	Baseline Night	Test Nights			
		*Air Fan*	*Buzzer*	*Vibrating Pillow*	*Fire Alarm*
**Young adults (11)**	768	207	158	170	17
**Children (4)**	256	191	66	92	12

**Table 3:** Effects of the device on young adults and children in the test nights versus the baseline night [Median (IQR)].

Young Adults			
	Test Nights (T_Ns_)	Baseline Night (B_N_)	*p*
**% awakenings**	24.5 (21)%	5.6 (9.4)%	**0.018**
**Sleep efficiency**	71.3 (7.2)%	79.3 (10.8)%	0.153
**% arousals**	23 (23.4)%	0 (1.3)%	0.180
**Risk time (s)**	4 (7)	9 (31)	**<0.001**
**Children**			
	**Test Nights (** **T_Ns_)**	**Baseline Night (** **B_N_)**	***p***
**% awakenings**	10.7 (7.4)%	3.3 (5.4)%	0.180
**Sleep efficiency**	88.2 (9)%	88.2 (11)%	0.593
**% arousals**	29 (15.1)%	0 (1.3)%	0.109
**Risk time (s)**	6 (11)	3 (8)	**<0.001**

**Table 4:** Effects of the device on young adults and children broken down by the type of triggered actuator [Median (IQR)].

Young Adults					
	Air Fan	Buzzer	Vibrating Pillow	Fire Alarm	*p*
**% awakenings**	36.7 (65.5)%	23.3 (67)%	38.9 (51.5)%	100 (57)%	0.664
**% arousals**	19.2 (21)%	12.5 (46.1)%	0 (6.8)%	66.7 (50)%	0.482
**Risk time (s)**	4 (4.5)	6.5 (7)	4.5 (7)	1.5 (13.5)	0.366
**Children**					
	**Air Fan**	**Buzzer**	**Vibrating Pillow**	**Fire Alarm**	***p***
**% awakenings**	13.6 (16.7)%	0 (3.7)%	18.8 (16.7)%	0 (0)%	0.223
**% arousals**	39.2 (21)%	2 (4.5)%	4.1 (15.4)%	66.7 (25)%	0.117
**Risk time (s)**	6 (11)	7 (20.25)	6 (11)	33 (0)	0.420

## Data Availability

The data presented in this study are available upon request from the corresponding author.
